# Association of metabolic syndrome and sarcopenia with all-cause and cardiovascular mortality: a prospective cohort study based on the NHANES

**DOI:** 10.3389/fendo.2024.1346669

**Published:** 2024-03-26

**Authors:** Weihong Huang, Siyi Deng, Siyang Liu, Qintao Ma, Liting Cao, Lan Liu, Heng Wan, Jie Shen

**Affiliations:** ^1^ Department of Endocrinology and Metabolism, Shunde Hospital, Southern Medical University (The First People’s Hospital of Shunde), Foshan, Guangdong, China; ^2^ Department of Cardiology, The First Affiliated Hospital of Shantou University Medical College, Shantou, Guangdong, China

**Keywords:** metabolic syndrome, sarcopenia, all-cause mortality, cardiovascular mortality, NHANES

## Abstract

**Background:**

Metabolic syndrome (MetS) and sarcopenia (SP) have emerged as significant public health concerns in contemporary societies, characterized by shared pathophysiological mechanisms and interrelatedness, leading to profound health implications. In this prospective cohort study conducted within a US population, we aimed to examine the influence of MetS and SP on all-cause and cardiovascular mortality.

**Methods:**

This study analyzed data from the National Health and Nutrition Examination Survey (NHANES) III for the years 1999-2006 and 2011-2018, and death outcomes were ascertained by linkage to National Death Index (NDI) records through December 31, 2019. Cox proportional hazard models were used to estimate hazard ratios (HRs) and 95% confidence intervals (95% CIs) for all-cause and cardiovascular mortality. In addition, subgroup and sensitivity analyses were conducted to test the robustness of the results.

**Results:**

Over a median follow-up period of 13.3 years (95% CI: 12.8-13.8), 1714 deaths were observed. The groups characterized by MetS−/SP+, MetS+/SP−, and MetS+/SP+ exhibited higher all-cause mortality rates in comparison to the MetS-/SP- group, with the MetS+/SP+ group (HR 1.76, 95% CI: 1.37-2.25) displaying the highest all-cause mortality. Increased cardiovascular mortality was observed in the MetS+/SP− (HR 1.84, 95% CI: 1.24-2.72), and MetS+/SP+ groups (HR 2.39, 95% CI: 1.32-4.35) compared to the MetS−/SP− group, whereas it was not statistically significant in the MetS-/SP+ group. However, among males and individuals aged < 60, the presence of both MetS and SP (MetS+/SP+ group) was found to be significantly associated with a higher risk of all-cause and cardiovascular mortality.

**Conclusion:**

The coexistence of MetS and SP increased the risk of all-cause and cardiovascular mortality, particularly in males and in nonelderly populations. Individuals with either MetS or SP may require more careful management to prevent the development of other diseases and thereby reduce mortality.

## 1 Introduction

Metabolic syndrome (MetS) is a group of clinical syndromes characterized by the aggregation of multiple disease states such as abdominal obesity, hypertension, dyslipidemia, abnormal glucose metabolism, and hyperuricemia in an individual (1). According to the National Health and Nutrition Examination Survey (NHANES), the prevalence of MetS has increased dramatically among U.S. adults, from 25.3% in 1988-1994 to 36.9% in 2015-2016 (2, 3). Most studies have shown that individuals with MetS have higher cardiovascular disease morbidity and mortality (4–6). Another study showed that MetS and its components were associated with all-cause, cardiovascular disease (CVD), and diabetes mortality (7).

Age-related reductions in skeletal muscle mass and strength, and diminished physical function are known as sarcopenia (SP) (8). In an aging society, the prevalence of SP is increasing globally, with an overall prevalence of 10-27% (9). Most studies have shown that individuals in SP are associated with a high risk of all-cause mortality (10–12).

Insulin resistance and chronic inflammation, as pathophysiological mechanisms common to both MetS and SP, interact to produce deleterious metabolic effects (13–17). MetS increases the risk of physical capacity and dysfunction (18–20) and is associated with lower muscle mass and strength (21). A meta-analysis demonstrated a positive association between SP and MetS (odds ratio, OR 2.01, 95% CI, 1.63-2.47) (22). MetS and SP are thought to be bi-directionally linked, increasing the risk of mutual morbidity (22, 23). Current studies on the comorbidity of MetS and SP have focused on the risk of cardiovascular disease, diabetes, and hyperlipidemia (24), and no studies comprehensively analyze the association of MetS and SP with mortality.

MetS and SP are highly prevalent worldwide and pose a significant public health burden. It may be valuable to assess the impact of their interaction on mortality in the general population. Therefore, this study investigates the association of MetS and SP with all-cause and cardiovascular mortality among U.S. adults using a sample that is nationally representative of the U.S. population.

## 2 Materials and methods

### 2.1 Study design and participants

Data for the study were obtained from the NHANES III, a research program conducted by the National Center for Health Statistics (NCHS), Centers for Disease Control and Prevention designed to assess the health and nutritional status of adults and children in the U.S. NHANES utilizes a complex, multistage, probability sampling approach to obtain data through questionnaires, interviews, mobile medical examinations, and laboratory tests (25). The NHANES data are free and available on the Web (26).

This study was conducted in accordance with the Declaration of Helsinki (27). Written informed consent was obtained from all study participants, and the program was approved by the Ethics Review Board of the National Center for Health Statistics (28).

**Figure 1:**
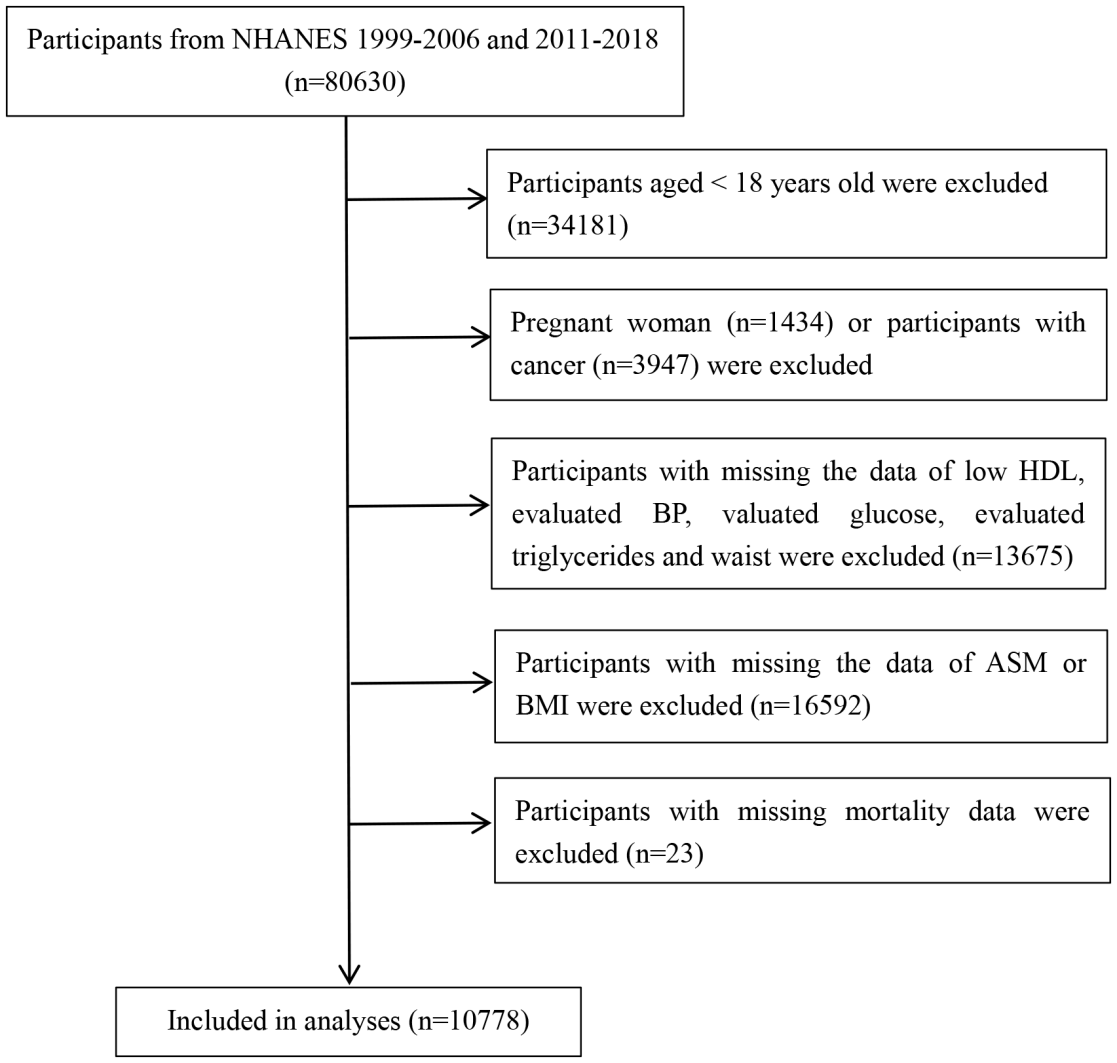
Study flowchart displaying the selection of patients according to exclusion criteria. BP, blood pressure; HDL, high-density lipoprotein cholesterol; ASM, appendicular skeletal muscle mass.

This study analyzed data from 1999-2006 and 2011-2018 and included 80,630 participants. Participants who were aged < 18 years, pregnant females, had a history of cancer at the time of enrollment, missing data on metabolic syndrome-related components, including blood pressure (BP), fasting blood glucose (FBG), triglycerides (TG), high-density lipoprotein cholesterol (HDL-C), and waist circumference (WC), missing data on the skeletal muscle mass of the extremities, and missing data on mortality were excluded, and 10,778 subjects were ultimately included in the study analysis. The flow chart of the study is shown in **Figure 1**.

### 2.2 Ascertainment of MetS and SP

MetS was defined according to the NCEP ATP III-2005 criteria (29). People with three or more of the following criteria were diagnosed with MetS: (1) elevated WC (EWC): WC ≥ 102 cm in men and ≥ 88 cm in women; (2) elevated BP (EBP): BP ≥ 130/85 mm Hg or drug treatment of previously diagnosed hypertension; (3) reduced HDL-C (RHDL-C): < 40 mg/dL in men and < 50 mg/dL in women or specific treatment for reduced HDL-C; (4) elevated TG (ETG): TG level ≥ 150 mg/dL or drug treatment for elevated TG; and (5) elevated fasting glucose (EGLU): fasting glucose level of ≥100 mg/L or drug treatment for elevated glucose and previously diagnosed type 2 diabetes. The unit of HDL-C converted to mmol/L is equal to mg/dL*0.0259. The unit of TG converted to mmol/L is equal to mg/dL* 0.0113. The unit of FBG converted to mmol/L is equal to mg/dL*18.

Appendicular skeletal muscle mass (ASM), the sum of the lean mass of extremities, was assessed using dual-energy X-ray (DXA) (QDR Discovery; Hologic, Inc., Bedford, MA, USA). In this study, SP used ASM divided by body mass index (BMI) (ASM/BMI) with cutoff points of ≤ 0.789 in men and ≤ 0.512 in women according to the Foundation for the National Institutes of Health (FNIH) criteria, which widely used in recent research (30).

Based on these definitions, the participants were categorized into the following four groups according to the presence of MetS and SP: 1) without MetS or SP (MetS−/SP−), 2) with MetS but no SP (MetS+/SP−), 3) without MetS but with SP (MetS−/SP+), and 4) with both MetS and SP (MetS+/SP+).

### 2.3 Ascertainment of covariates

Study data also included sex, age, race and ethnicity, smoking status, drinking status, physical activity, marital status, education level, family poverty-to-income ratio (PIR), height, and weight. Participants’ race and ethnicity were categorized into four groups: Mexican American, non-Hispanic White, non-Hispanic Black, or others (31). Never smokers were defined as those who smoked fewer than 100 cigarettes in their lifetime, those who smoked at least 100 cigarettes in their lifetime were categorized as current smokers, and those who smoked at least 100 cigarettes in their lifetime and quit were labeled ex-smokers (32). Alcohol consumption was determined by a cutoff of ≥ 12 drinks per year, with no alcohol consumption defined as drinking < 12 drinks per year (33). Ideal physical activity was defined as at least 150 minutes of moderate or 75 minutes of vigorous physical activity per week, according to US PA guidelines (34). Educational level was categorized as less than high school, high school or equivalent, college or above, and marital status was categorized as married, separated, including widowed and divorced groups, or never married (7). Family PIR levels were grouped into three categories: 0-1.0, 1.1-3.0, and > 3.0 (35). BMI was calculated as weight (kg) divided by height squared (m^2^). Multiple interpolation was used for missing values of covariates.

### 2.4 Ascertainment of death

Mortality status was ascertained by probabilistic matching to the NDI through December 31, 2019, using a unique study identifier. Details of the matching method are available from the NCHS (36). Causes of death were classified according to the codes of ICD-10. Primary outcomes in this study were mortality from all causes, heart diseases (codes I00-I09, I11, I13, and I20-I51).

### 2.5 Statistical analysis

In accordance with the NHANES analysis guidelines, all analyses considered a complex survey design, including sample weights, clustering, and stratification. Missing values for covariates were supplemented using multiple interpolations. Continuous variables were expressed as weighted mean ± standard deviation for weighted characterization, while categorical variables were expressed as frequencies with weighted percentages. Differences in covariates across the groups were performed using one-way ANOVA for continuous variables and the Rao-Scott chi-square test for categorical variables with adjusted weights. Survival curves associated with all-cause and cardiovascular mortality were plotted according to the presence of MetS and SP using the Kaplan-Meier method. Cox proportional hazards models were used to calculate hazard ratios (HRs) and their 95% confidence intervals (95% CIs) for all-cause mortality and cardiovascular mortality, and *P* values for trends were calculated. Model 1 was unadjusted, Model 2 was adjusted for age, sex, and race/ethnicity, and Model 3 was adjusted for age, sex, race/ethnicity, education level, marital status, family PIR, smoking status, drinking status, and physical activity. We also performed stratified analyses by sex and age, obtaining *P* values for interactions. Cox regression analysis was performed in the SP population according to the number of abnormal metabolic components and the type of metabolic abnormality. To test the robustness of the findings, we performed three sensitivity analyses: first, we excluded participants with prior myocardial infarction or angina; second, we excluded participants with a prior history of stroke; and finally, we excluded participants who died within 2 years of the follow-up period.

All statistical analyses were performed using R (version 4.2.3; R Foundation for Statistical Computing) to account for the NHANES complex sample design, with 2-sided *P* < 0.05 considered statistically significant.

## 3 Results

### 3.1 Baseline characteristics

**Table 1** summarizes the baseline characteristics of the four groups of subjects. Among the 10,778 subjects, 6,962 (64.6%) had neither MetS nor SP (MetS−/SP−), 486 (4.5%) had SP only (MetS−/SP+), 2693 (25.0%) had MetS only (MetS+/SP−), and 637 (5.9%) had both MetS and SP (MetS+/SP+). Subjects in the MetS+/SP+ group were significantly older than those in the other groups (*P* < 0.001). Subjects in the MetS+/SP+ group had higher all-cause mortality (25.2%) and cardiovascular mortality (7.2%). BMI, WC, TG, and FBG in the MetS+/SP+ group were also significantly higher than those in the other groups (*P* < 0.001). Differences between groups were significant except for the sex group.

**Table 1:** Baseline characteristics of the study subjects.

	Mets-/SP- (n=6962)	Mets-/SP+ (n=486)	Mets+/SP- (n=2693)	Mets+/SP+ (n=637)	*P* value
**Age(years)**	37.06 ± 13.21	45.74 ± 16.49	47.26 ± 12.99	52.95 ± 14.47	<0.001
**Sex (%)**					0.210
**Male**	3633(51.4)	266(55.6)	1295(50.2)	326(55.2)	
**Female**	3329(48.6)	220(44.4)	1398(49.8)	311(44.8)	
**Race and ethnicity (%)**					<0.001
**Mexican American**	1253(9.0)	199(19.8)	482(7.7)	297(21.6)	
**Non-Hispanic White**	1359(14.3)	110(21.6)	419(11.8)	90(15.7)	
**Non-Hispanic Black**	2669(64.5)	159(56.5)	1164(69.2)	217(59.7)	
**Other races**	1681(12.3)	18(2.1)	628(11.3)	33(3.0)	
**Alcohol consumption (%)**					0.005
**Yes**	3090(46.8)	201(42.5)	1123(42.2)	247(39.3)	
**No**	3872(53.2)	285(57.5)	1570(57.8)	390(60.7)	
**Smoking status (%)**					<0.001
**Never smoker**	4062(56.1)	298(61.2)	1359(49.3)	334(50.8)	
**Ever smoker**	1252(19.9)	111(20.2)	692(26.5)	192(28.0)	
**Current smoker**	1648(24.0)	77(18.7)	642(24.2)	111(21.2)	
**Ideal physical activity (%)**					0.006
**Yes**	3594(50.2)	228(50.4)	1221(45.5)	313(47.4)	
**No**	3368(49.8)	258(49.6)	1472(54.5)	324(52.6)	
**Married status (%)**					<0.001
**married**	3090(51.0)	262(55.9)	1478(59.1)	380(62.4)	
**separated**	873(12.1)	93(15.6)	583(19.3)	148(20.0)	
**never married**	2999(36.9)	131(28.5)	633(21.7)	109(17.6)	
**Educational levels (%)**					<0.001
**less than high school**	1556(15.3)	213(31.6)	739(18.0)	311(30.2)	
**high school or equivalent**	1545(22.0)	105(25.8)	651(27.4)	141(28.1)	
**college or above**	3861(62.7)	168(42.5)	1303(54.5)	185(41.7)	
**Family poverty-to-income ratio level (%)**					<0.001
**0-1.0**	1591(15.3)	132(21.4)	533(13.9)	180(21.2)	
**1.1-3.0**	2716(35.3)	223(42.0)	1149(37.8)	310(46.1)	
**>3.0**	2655(49.4)	131(36.6)	1011(48.3)	147(32.7)	
**BMI(kg/m2)**	26.16 ± 5.38	30.80 ± 6.31	32.38 ± 6.24	35.41 ± 7.22	<0.001
**Waist(cm)**	90.56 ± 13.50	101.54 ± 15.12	108.34 ± 14.02	114.09 ± 15.44	<0.001
**TG(mmol/L)**	1.13 ± 0.74	1.33 ± 0.66	2.25 ± 1.91	2.26 ± 1.95	<0.001
**HDL(mmol/L)**	1.45 ± 0.39	1.41 ± 0.37	1.16 ± 0.32	1.18 ± 0.34	<0.001
**FBG(mmol/L)**	5.28 ± 0.83	5.65 ± 1.66	6.51 ± 2.33	7.07 ± 2.74	<0.001
**ABP(mmHg)**	115.57 ± 13.54	122.16 ± 17.46	128.18 ± 16.83	132.97 ± 18.38	<0.001
**DBP(mmHg)**	69.07 ± 10.61	71.34 ± 11.57	75.69 ± 12.94	73.74 ± 14.07	<0.001
**All-cause mortality (%)**					<0.001
**Yes**	408(4.6)	106(16.8)	431(11.9)	204(25.2)	
**No**	6554(95.4)	380(83.2)	2262(88.1)	433(74.8)	
**Cardiovascular mortality (%)**					<0.001
**Yes**	93(0.9)	29(4.1)	135(3.3)	56(7.2)	
**No**	6869(99.1)	457(95.9)	2558(96.7)	581(92.8)	

Data are shown as the mean ± SD or frequency (percentage). SD, standard deviation; BMI, body mass index; TG, triglyceride; HDL, high-density lipoprotein cholesterol; FBG, fasting blood glucose; ABP, arterial blood pressure; DBP, diastolic blood pressure; MetS, metabolic syndrome; SP, sarcopenia.

### 3.2 Association of MetS and SP status with mortality

**Figure 2:**
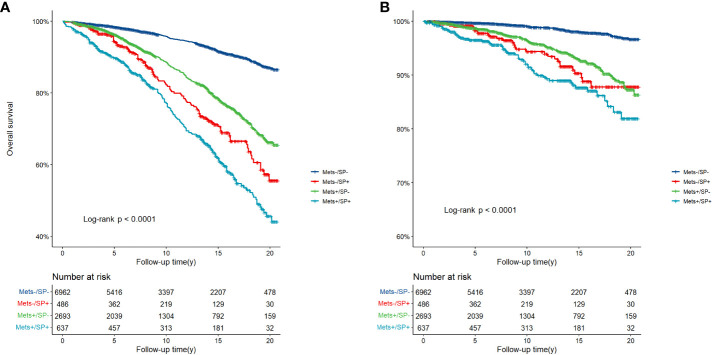
**(A)** Overall survival, and **(B)** CVD-related survival according to MetS and SP status. CVD, cardiovascular disease; MetS, metabolic syndrome; SP, sarcopenia.

There were 1714 deaths during the follow-up period: 1149 (10.66%) from all-cause mortality and 313 (2.90%) from CVD. **Figure 2** depicts the survival curves of the four groups of subjects and shows significant differences in overall survival and cardiovascular survival among the groups during a median follow-up duration of 13.3 years (95% CI: 12.8-13.8) (three-group log-rank *P* < 0.001). The MetS−/SP− group had the best survival, whereas the MetS+/SP+ group had the worst survival among all the groups.

**Table 2** shows the association of MetS and SP status with all-cause and cardiovascular mortality. After adjusting for sex, age, race and ethnicity, smoking status, drinking status, physical activity, marital status, education level, and PIR, compared to the MetS−/SP− group, the risk of all-cause mortality was increased in the MetS−/SP+ group (HR 1.52, 95% CI: 1.15-2.01, *P* = 0.003), the MetS+/SP− group (HR 1.32, 95% CI: 1.06-1.64, *P* = 0.012), and the MetS+/SP+ group (HR 1.76, 95% CI: 1.37-2.25, *P* < 0.001). There was an increased risk of cardiovascular death in the MetS+/SP− (HR 1.84, 95% CI: 1.24-2.72, *P* = 0.002) and MetS+/SP+ (HR 2.39, 95% CI: 1.32-4.35, *P* = 0.004) groups compared with the MetS−/SP− group, whereas there was no significant difference in the MetS−/SP+ group. The HRs for all-cause mortality gradually increased in the MetS−/SP−, MetS+/SP−, MetS−/SP+, and MetS+/SP+ groups (*P* for trend < 0.001). A similar trend was observed for cardiovascular mortality (*P* for trend < 0.001).

**Table 2:** Risks of all-cause and cardiovascular mortality according to the presence of MetS or SP status.

	MetS-/SP-	MetS-/SP+	*P v*alue	MetS+/SP-	*P* value	MetS+/SP+	*P* value	*P* for trend
	HR	HR(95%CI)		HR(95%CI)		HR(95%CI)		
All-cause mortality								
**Model 1**	1 (ref)	4.41 (3.18,6.11)	<0.001	2.65 (2.19,6.11)	<0.001	6.27 (4.93,7.98)	<0.001	<0.001
**Model 2**	1 (ref)	1.57 (1.18,2.09)	0.002	1.33 (1.09,1.62)	0.006	1.89 (1.46,2.44)	<0.001	<0.001
**Model 3**	1 (ref)	1.52 (1.15,2.01)	0.003	1.32 (1.06,1.64)	0.012	1.76 (1.37,2.25)	<0.001	<0.001
Cardiovascular mortality								
**Model 1**	1 (ref)	5.64 (2.84,11.18)	<0.001	3.88 (2.65,5.66)	<0.001	9.59 (5.58,16.50)	<0.001	<0.001
**Model 2**	1 (ref)	1.60 (0.80,3.20)	0.188	1.82 (1.26,2.63)	0.002	2.469 (1.41,4.34)	0.002	<0.001
**Model 3**	1 (ref)	1.53 (0.79,2.96)	0.212	1.84 (1.24,2.72)	0.002	2.39 (1.32,4.35)	0.004	<0.001

Model 1: unadjusted.

Model 2: adjusted for age, sex, and race.

Model 3: adjusted for age, sex, race, physical activity, alcohol consumption, smoking status, educational levels, marital status, and family poverty-to-income ratio.

MetS, metabolic syndrome; SP, sarcopenia; HR, hazard ratio; ref, reference.

**Table 3** presents the association of MetS and its components with all-cause and cardiovascular mortality in general and SP populations. After adjusting for covariates, when the number of metabolic abnormalities was ≥ 4, there was a significant positive association between the number of MetS components and all-cause and cardiovascular mortality. As the number of MetS components increased, the risk of all-cause and cardiovascular mortality increased. After adjusting for covariates, all five components of MetS were associated with an increased risk of all-cause and cardiovascular mortality. Only when the number of metabolic abnormalities was equal to 4 (HR 2.38, 95% CI: 1.51-4.92, *P* = 0.019) was associated with cardiovascular mortality in the SP population, and no other number of metabolic abnormalities was observed to be associated with all-cause and cardiovascular mortality (*P* > 0.05). Only ETG (HR 1.42, 95% CI: 1.00-2.00, *P* = 0.046) was associated with all-cause mortality, whereas the other metabolic abnormality components were not associated with all-cause and cardiovascular mortality.

**Table 3:** Associations of MetS and its components with all-cause and cardiovascular mortality in the general population and in the SP population.

	All-cause mortality	*P* value	Cardiovascular mortality	*P* value
	HR (95% CI)		HR (95% CI)	
In the general population				
Number of MetS				
**< 3**	1 (ref)		1 (ref)	
**3**	1.04(0.80,1.36)	0.761	1.22(0.81,1.84)	0.343
**4**	1.57(1.27,1.94)	<0.001	2.38(1.65,3.44)	<0.001
**5**	1.65(1.24,2.20)	<0.001	2.49(1.39,4.46)	0.002
***P* for trend**		<0.001		<0.001
Components of MetS				
**EBP**	1.30(1.09,1.56)	0.004	1.96(1.35,2.84)	<0.001
**EGLU**	1.27(1.09,1.47)	0.003	1.55(1.11,2.15)	0.009
**ETG**	1.27(1.10,1.47)	<0.001	1.38(1.05,1.81)	0.022
**RHDL-C**	1.35(1.12,1.61)	0.001	1.47(1.01,2.14)	0.043
**EWC**	1.35(1.10,1.67)	0.005	1.84(1.37,2.47)	<0.001
In the SP population				
Number of MetS				
**< 3**	1 (ref)		1 (ref)	
**3**	1.10(0.68,1.77)	0.706	1.22(0.47,3.16)	0.681
**4**	1.31(0.78,2.21)	0.311	2.38(1.15,4.92)	0.019
**5**	1.28(0.79,2.10)	0.318	1.03(0.42,2.53)	0.943
***P* for trend**		0.216		0.117
Components of MetS				
**EBP**	1.08(0.72,1.63)	0.71	1.79(0.77,4.16)	0.175
**EGLU**	1.09(0.74,1.59)	0.66	1.23(0.65,2.34)	0.539
**ETG**	1.42(1.00,2.00)	0.046	1.28(0.73,2.23)	0.388
**RHDL-C**	1.05(0.74,1.49)	0.798	1.23(0.64,2.37)	0.531
**EWC**	1.29(0.96,1.73)	0.088	1.64(0.75,3.58)	0.215

Model adjusted for age, sex, race, physical activity, alcohol consumption, smoking status, educational levels, marital status, and family poverty-to-income ratio. EBP, elevated blood pressure; EGLU, elevated fasting glucose; ETG, elevated triglycerides; RHDL-C, reduced high-density lipoprotein cholesterol; EWC, elevated waist circumference; ref, reference.

### 3.3 Subgroup analysis

The results of subgroup analyses of MetS and SP status with all-cause and cardiovascular mortality by sex and age are shown in **Table 4**. Compared to the MetS−/SP− group, the MetS+/SP+ group had a higher risk of all-cause mortality (HR 2.23, 95% CI: 1.53-3.24, *P* < 0.001) and cardiovascular mortality (HR 3.29, 95% CI: 1.37-7.88, *P* = 0.008) among the males, while no similar situation was observed in the females. Individuals aged < 60 years with MetS and SP had a higher risk of all-cause mortality (HR 4.48, 95% CI: 2.64-7.63, *P* < 0.001) and cardiovascular mortality (HR 8.88, 95% CI: 2.84-27.80, *P* < 0.001), whereas individuals aged ≥ 60 years with MetS and SP had higher hazard of all-cause mortality (HR 1.67, 95% CI: 1.32-2.10, *P* < 0.001), and cardiovascular mortality (HR 1.95, 95% CI: 1.07-3.53, *P* = 0.028).

**Table 4:** Risks of all-cause and cardiovascular mortality according to the presence of MetS or sarcopenia status, stratified by sex and age.

	All-cause mortality		*P f*or interaction	Cardiovascular mortality		*P* for interaction
	HR (95%CI)	*P* value		HR (95%CI)	*P* value	
Sex			0.001			0.806
Male^a^						
MetS-/SP-	1 (ref)			1 (ref)		
MetS-/SP+	1.72 (1.27,2.32)	<0.001		2.204 (0.91,4.95)	0.056	
MetS+/SP-	1.28 (0.97,1.68)	0.080		2.391 (1.42,4.01)	<0.001	
MetS+/SP+	2.23 (1.53,3.24)	<0.001		3.29 (1.37,7.88)	0.008	
Female^a^						
MetS-/SP-	1 (ref)			1 (ref)		
MetS-/SP+	1.31 (0.83,2.06)	0.251		0.92 (0.28,2.98)	0.882	
MetS+/SP-	1.36 (0.99,1.86)	0.053		1.28 (0.74,2.22)	0.373	
MetS+/SP+	1.26 (0.85,1.87)	0.261		1.56 (0.60,4.06)	0.367	
Age, years			<0.001			<0.001
<60 years^b^						
MetS-/SP-	1 (ref)			1 (ref)		
MetS-/SP+	2.04 (1.01,4.12)	0.047		2.97 (0.55,16.14)	0.208	
MetS+/SP-	2.00 (1.44,2.78)	<0.001		3.76 (1.85,7.69)	<0.001	
MetS+/SP+	4.48 (2.64,7.63)	<0.001		8.88 (2.84,27.80)	<0.001	
≥60 years^b^						
MetS-/SP-	1 (ref)			1 (ref)		
MetS-/SP+	1.90 (1.28,2.81)	0.001		1.85 (0.96,3.58)	0.068	
MetS+/SP-	1.33 (1.04,1.70)	0.024		1.47 (0.98,2.20)	0.064	
MetS+/SP+	1.67 (1.32,2.10)	<0.001		1.95 (1.07,3.53)	0.028	

Model^a^: adjusted for age, race, physical activity, alcohol consumption, smoking status, educational levels, marital status, and family poverty-to-income ratio.

Model^b^: adjusted for sex, race, physical activity, alcohol consumption, smoking status, educational levels, marital status, and family poverty-to-income ratio. MetS, metabolic syndrome; SP, sarcopenia; HR, hazard ratio; ref, reference.

### 3.4 Sensitivity analysis

Sensitivity analysis was performed by excluding the subjects with a previous myocardial infarction or angina (**Supplementary Table 1**). Compared to the MetS−/SP− group, the MetS+/SP+ group had an increased risk of all-cause mortality (HR 1.60, 95% CI: 1.21-2.10, *P* < 0.001) and cardiovascular mortality (HR 2.37, 95% CI: 1.17-4.80, *P* = 0.016). Subjects with SP only (MetS−/SP+ group) had only an increased risk of all-cause mortality (HR 1.57, 95% CI: 1.14-2.15, *P* = 0.005). Subjects with MetS only (MetS+/SP− group) had an increased risk of cardiovascular mortality (HR 1.72, 95% CI: 1.13-2.62, P = 0.012).

Sensitivity analysis was performed by excluding the subjects with a previous episode of stroke (**Supplementary Table 2**). All-cause mortality was significantly increased in the MetS−/SP+ (HR 1.55, 95% CI: 1.16-2.06, *P* = 0.003), MetS+/SP− (HR 1.31, 95% CI: 1.06-1.62, *P* = 0.013), and MetS+/SP+ (HR 1.70, 95% CI: 1.31-2.20, *P* < 0.001) groups compared with the MetS−/SP− group (*P* for trend < 0.001). Cardiovascular mortality was increased in the MetS+/SP− group (HR 1.79, 95% CI: 1.20-2.66, *P* = 0.004) and the MetS+/SP+ group (HR 2.38, 95% CI: 1.25-4.53, *P* = 0.008), but statistical significance was not reached in the MetS−/SP+ group.

Sensitivity analysis was performed by excluding the subjects who had died within two years of follow-up (**Supplementary Table 3**). Similarly, all-cause mortality was significantly increased in the MetS−/SP+ (HR 1.52, 95% CI: 1.09-2.11, P = 0.013), MetS+/SP− (HR 1.33, 95% CI: 1.07-1.67, *P* = 0.011), and MetS+/SP+ (HR 1.63, 95% CI: 1.26-2.11, *P* < 0.001) groups compared with the MetS−/SP− group (*P* for trend < 0.001). Cardiovascular mortality was significantly increased in the MetS+/SP− group (HR 1.87, 95% CI: 1.28-2.73, *P* = 0.001) and MetS+/SP+ group (HR 2.36, 95% CI: 1.30-4.31, *P* = 0.005).

## 4 Discussion

Our study retrospectively assessed the association of MetS and SP with all-cause and cardiovascular mortality mortality. In our study, the coexistence of MetS and SP was independently and positively associated with an elevated risk of all-cause and cardiovascular mortality after adjusting for potential confounders such as sociodemographic factors, lifestyle factors, and other factors. Mortality risks almost doubled in the MetS and SP coexistence group (HR 1.76 for all-cause mortality, HR 2.39 for cardiovascular mortality), and such a trend was observed in males (HR 2.23 for all-cause mortality, HR 3.29 for cardiovascular mortality) and nonelderly individuals (HR 4.48 for all-cause mortality, HR 8.88 for cardiovascular mortality) more significantly. Our findings suggest that the coexistence of MetS and SP increases the risk of all-cause and cardiovascular mortality, especially in male and nonelderly populations.

In our study, 56.7% (637/1123) of patients with SP showed coexistence with MetS, and 19.1% (637/3330) of patients with MetS showed coexistence with SP. The reasons for this phenomenon may be related to some common pathogenesis between the two (13–17). Several features of MetS may damage muscle health, including insulin resistance and chronic systemic inflammation, which negatively affects muscle homeostasis, leading to reduced muscle mass and strength (14, 37–39), and oxidative stress leading to mitochondrial dysfunction and impaired muscle repair of damage (39, 40), which in turn leads to decreased muscle function (4). Skeletal muscle, as the largest organ in the body, plays a crucial role in maintaining glucose homeostasis and regulating carbohydrate metabolism (41). Low muscle mass may impair blood glucose uptake by altering myokine secretion, leading to a state of insulin resistance and increasing the degree of localized inflammation and metabolic disturbances, which may facilitate the development of MetS (42–44). MetS and SP may contribute to each other’s development based on the mechanisms described above. As the incidence and prevalence of both MetS and SP are rapidly increasing in current society, the bidirectional relationship between these diseases may lead to amplified health risks in the population, and it is meaningful to study the mortality risk of MetS and SP comorbidity for an aging society.

Our study shows that all-cause and cardiovascular mortality was higher in the MetS and SP coexistence group than in the group with one disease alone, whereas there was no significant difference in the SP-only group compared with the standard control group, which is similar to the findings of Eyun Song et al. (4). The impact of MetS on cardiovascular mortality is higher than that of SP, which may be due to the common pathogenesis between MetS and CVD (4–6). MetS and SP may contribute to each other’s disease progression through some mechanism, thus increasing the risk of death. However, the possible mechanisms leading to this situation need to be explored and verified by more basic and clinical studies.

Our study shows that the effect of the state of presence of MetS and SP on all-cause and cardiovascular mortality is not identical in different populations. In the male population, all-cause and cardiovascular mortality was significantly higher in the group in which MetS and SP coexisted and was higher than in the group in which MetS or SP alone was present. In contrast, we did not observe this trend in the female population. For the nonelderly population (< 60 years), this trend was also evident, however, in the elderly population (≥ 60 years), the group with the coexistence of MetS and SP had significantly higher cardiovascular mortality than the group with only one disease. Previous studies have shown that the adverse effects of MetS on muscle mass and strength are mainly seen in young males. However, females are mostly less susceptible to the adverse effects of MetS on muscle (42). This is similar to the results of the present study, where this trend of having a higher risk of death when MetS and SP coexisted was more pronounced in male and nonelderly populations. The mechanism responsible for this phenomenon may be related to the effects of adipokines on skeletal muscle (45), which are produced and released by adipose tissue, such as lipocalin, leptin, and proinflammatory cytokines (46). Skeletal muscle is a crucial target tissue affected by these molecules, and their circulation levels are influenced by age and sex (47). In young women, serum lipocalin and leptin do not appear to be significantly associated with skeletal muscle morphology and function (48). However, in males, skeletal muscle seems more vulnerable to the impact of adipokines. Another possible explanation could be sex hormones, with MetS being associated with reduced testosterone levels (49), and testosterone being positively correlated with muscle strength (50). Since testosterone levels decrease with age (51) and women have lower testosterone levels than men, young men with relatively high testosterone levels may be particularly vulnerable.

Our research has several advantages. First, we adopted a prospective cohort study of a large, nationally representative sample. For the study population, we had a relatively adequate follow-up period and a reliable assessment of the causes of death of the study population. Second, a nationally representative community sample, standardized data collection procedures, and complete follow-up of survival times conducted by the U.S. government more than validate our study. In addition, we performed a detailed analysis of the association of MetS and SP presence status with all-cause and cardiovascular mortality, adjusting for a large number of potential confounders. The analyses were stratified to explore the effects of sex and age on the experimental results, and three sensitivity analyses were conducted to investigate the stability of the results. However, this study has several limitations. First, the results of this study may be representative of U.S. residents only, and the definition and cutoff value of SP varied by race. Therefore, the results cannot be generalized to the general population of different races and need to be further validated in other races. Second, alcohol consumption, smoking, and ideal physical activity were self-reported, which may not be accurate. Third, residual bias could not be eliminated despite adjusting for confounding mortality-related variables.

## 5 Conclusions

In summary, for US adults, MetS or SP is associated with a high risk of all-cause and cardiovascular mortality, and this relationship is more pronounced in males or nonelderly adults. MetS and SP as comorbidities increased the risk of all-cause and cardiovascular death compared with the presence of each condition alone. Future research is needed to reveal the mechanisms underlying the association between MetS, SP, and mortality and finding simple and practical criteria for screening patients with MetS and SP for early intervention is important for improving the healthy life expectancy of the population, which should be of concern to health care professionals.

## Data availability statement

The raw data supporting the conclusions of this article will be made available by the authors, without undue reservation.

## Ethics statement

The studies involving humans were approved by National Center for Health Statistics Institutional Review Board. The studies were conducted in accordance with the local legislation and institutional requirements. The participants provided their written informed consent to participate in this study.

## Author contributions

WH: Writing – review & editing, Writing – original draft, Software, Methodology, Data curation, Conceptualization. SD: Writing – review & editing, Writing – original draft, Software, Data curation. SL: Writing – review & editing, Methodology. QM: Writing – review & editing, Data curation. LC: Writing – review & editing, Data curation. LL: Writing – review & editing, Methodology. HW: Writing – review & editing, Supervision, Methodology. JS: Writing – review & editing, Supervision, Methodology.
