# Supplementary material for: Association of metabolic syndrome and sarcopenia with all-cause and cardiovascular mortality: a prospective cohort study based on the NHANES
Source: Front Endocrinol (Lausanne). 2024 Mar 26;15:1346669. doi: 10.3389/fendo.2024.1346669 (PMC11002088; doi:10.3389/fendo.2024.1346669)
Supplement: Supplementary file 1 [file Table_1.docx]

Supplementary Material

# Supplementary Data

# The NHANES data are free and available on the Web: https://wwwn.cdc.gov/nchs/nhanes/analyticguidelines.aspx

# Supplementary Tables

**Supplementary Table 1**  Adjusted HRs of MetS or SP status and all-causes and cardiovascular mortality, excluding the participants with a previous myocardial infarction or angina.

|  | **All-cause mortality** | ***P* value** | **Cardiovascular mortality** | ***P* value** |
| --- | --- | --- | --- | --- |
|  | **HR (95% CI)** |  | **HR (95% CI)** |  |
| **MetS-/SP-** | 1 (ref) |  | 1 (ref) |  |
| **MetS-/SP+** | 1.57(1.14,2.15) | 0.005 | 1.79(0.89,3.60) | 0.104 |
| **MetS+/SP-** | 1.25(0.99,1.59) | 0.066 | 1.72(1.13,2.62) | 0.012 |
| **MetS+/SP+** | 1.60(1.21,2.10) | <0.001 | 2.37(1.17,4.80) | 0.016 |
| ***P* for interaction** |  | 0.058 |  | 0.005 |

All adjusted for age, sex, race, physical activity, alcohol consumption, smoking status, educational levels, marital status, and family poverty-to-income ratio. Abbreviations: MetS, metabolic syndrome; SP, sarcopenia; HR, hazard ratio; ref, reference.

**Supplementary Table 2** Adjusted HRs of MetS or SP status and all-causes and cardiovascular mortality, excluding the participants with a previous episode of stroke.

|  | **All-cause mortality** | ***P* value** | **Cardiovascular mortality** | ***P* value** |
| --- | --- | --- | --- | --- |
|  | **HR (95% CI)** |  | **HR (95% CI)** |  |
| **MetS-/SP-** | 1 (ref) |  | 1 (ref) |  |
| **MetS-/SP+** | 1.55(1.16,2.06) | 0.003 | 1.58(0.76,3.28) | 0.217 |
| **MetS+/SP-** | 1.31(1.06,1.62) | 0.013 | 1.79(1.20,2.66) | 0.004 |
| **MetS+/SP+** | 1.70(1.31,2.20) | <0.001 | 2.38(1.25,4.53) | 0.008 |
| ***P* for interaction** |  | <0.001 |  | <0.001 |

All adjusted for age, sex, race, physical activity, alcohol consumption, smoking status, educational levels, marital status, and family poverty-to-income ratio. Abbreviations: MetS, metabolic syndrome; SP, sarcopenia; HR, hazard ratio; ref, reference.

**Supplementary Table 3** Adjusted HRs of MetS or SP status and all-causes and cardiovascular mortality, excluding who had died within 2 years of follow-up.

|  | **All-cause mortality** | ***P* value** | **Cardiovascular mortality** | ***P* value** |
| --- | --- | --- | --- | --- |
|  | **HR (95% CI)** |  | **HR (95% CI)** |  |
| **MetS-/SP-** | 1 (ref) |  | 1 (ref) |  |
| **MetS-/SP+** | 1.52(1.09,2.11) | 0.013 | 1.59(.82,3.08) | 0.175 |
| **MetS+/SP-** | 1.33(1.07,1.67) | 0.011 | 1.87(1.28,2.73) | 0.001 |
| **MetS+/SP+** | 1.63(1.26,2.11) | <0.001 | 2.36(1.30,4.31) | 0.005 |
| ***P* for interaction** |  | <0.001 |  | <0.001 |

All adjusted for age, sex, race, physical activity, alcohol consumption, smoking status, educational levels, marital status, and family poverty-to-income ratio. Abbreviations: MetS, metabolic syndrome; SP, sarcopenia; HR, hazard ratio; ref, reference.
